# Clinical and cost-effectiveness of synbiotics versus antibiotics and analgesics in post-tooth extraction socket healing: A randomized controlled trial

**DOI:** 10.6026/973206300211719

**Published:** 2025-05-31

**Authors:** Heena Shaikh, Lata Ajay Tapnikar, Vidyalakshmi Shankararaman, Shrikant Patel, Dhrumi Shah, Vyoma Patel

**Affiliations:** 1 Department Hospital & Healthcare Management, Trinity Western University, Canada; 2 Department of Community Medicine, GS Medical College, Hapur, India; 3 Private Practice, Sai Shankar Multispeciality Clinic, Arumbakkam Chennai, Humber College, Ontario, Canada; 4 Department of Cariology and Comprehensive Care, NYU College of Dentistry, New York, USA

**Keywords:** Antibiotics, analgesics synbiotics, tooth extraction

## Abstract

There has been a noticeable increase in the use of alternative strategies such as herbal medicines and laser therapies to tackle a range of health issues, including oral diseases. Therefore, it is of interest to examine how synbiotics affect tooth extraction socket healing and problems. Hence, a total of 210 patients, were split into three groups of 70 individuals each. The primary outcome measures were assessed on the 3rd, 5th, and 7th days after the extraction. Group 3 patients had the highest incidence of bleeding, while group 1 had the lowest. Group 3 experienced the highest level of discomfort and swelling, while group 1 reported the lowest. Thus, post-extraction wound healing using synbiotics' benefits.

## Background:

The human mouth contains four wisdom teeth, each positioned in a different location: upper left, upper right, lower left, and lower right. Impacted teeth, especially the lower M3, can increase the likelihood of surgical complications because of their abnormal position and blocked eruption. Recurrent pericoronitis of the third molar can occur as a result of this. Periodontitis represents an inflammatory pathology that impacts the supportive structures associated with the dentition in which the alveolar bone and the periodontal ligament gradually deteriorate as a result of this condition [1, 2, 3, 4, 5, 6, 7, 8- 9]. Higher proportion of males experiencing this sensation compared to females, warrants further investigation. While the underlying reasons for this gender disparity remain unclear, it may reflect variations in pain perception and reporting between genders, highlighting the importance of considering gender-specific factors in pain management approaches [10]. There has been a growing interest among scientists regarding the potential advantages of synbiotics in preserving oral health. A product with probiotics and prebiotics is "synbiotic". In this synergistic connection, the prebiotic component supports the probiotic compound [11].

Probiotics Studies indicate that synbiotics have the potential to lower the likelihood of developing infectious disorders such as diarrhoea, as well as oral conditions like dental caries, mouth ulcers, gingivitis, and periodontal diseases. Hull *et al.* (1984) introduced Lactobacillus acidophilus and Holcombh *et al.* (1991) identified Bifidobacterium bifidum. There is a lack of information in the literature when it comes to managing patients with oral diseases using synbiotics, despite the potential benefits they offer [12, 13]. Overusing antibiotics can result in antibiotic resistance, a pressing issue on a global scale. As someone passionate about advocating for the prevention of antibiotic resistance, I carefully select synbiotics to set them apart from other products [13, 14]. There are a wide range of options when it comes to synbiotics, with different forms available such as powder, liquid, gel, paste, granules, capsules, and sachets [15- 16]. Despite the promising potential of synbiotics in promoting oral health, there remains a significant gap in the literature regarding their management and efficacy in patients with oral diseases. Comprehensive clinical studies specifically targeting their role in oral health management are sparse. Therefore, it is of interest to investigate the potential of synbiotics in invasive dental procedures, with the goal of reducing the risk of specific infectious diseases and reducing the need for antibiotics.

## Materials and Methods:

Received Institute Institutional Ethics Committee approval. (575/Ethics/GRIDC/2024) A formula determined sample size for accuracy and reliability. In this randomised clinical study, eligible participants were recruited and then assigned to one of three experimental groups using a computer-generated list.

## Inclusion criteria:

Nonsmokers and non-systemic medicine users no recent or ongoing antibiotic use.

## Exclusion criteria:

Before the surgery, it is important to identify any potential dental issues that may be present by examining the radiographs. Regular consumers of probiotics and prebiotics. There were three groups, each consisting of 70 patients. To determine the appropriate sample size, the formula used is n= z2pq/d2. A total of 210 people were included in the sample size. When the value of "n" reached 205, it was rounded up to 210 after calculation. All the necessary pre-operative information for each patient was carefully documented in the data sheet. This included the patient's age, gender, reasons for extraction (diagnosed through clinical and radiographic examinations), and the specific tooth or teeth that needed to be removed. Patients who had undergone the extraction of a multi-rooted tooth were included in the study. Patients in this study were provided with synbiotics in sachet form and were advised to follow the instructions on the packaging. The participants were randomly assigned to three different groups. Group 1 received synbiotics (Bifidobacterium longum + lactoferrin, ProBiora Health) in addition to analgesics as needed for a period of seven days. Group 2 received antibiotics and analgesics as needed, while Group 3 was only given analgesics as needed, without any antibiotics or synbiotics provided after the extraction. Following the extraction, we evaluated days 1, 4, and 7 to determine if any complications arose, such as bleeding, discomfort, swelling, and changes in gut flora. According to a study conducted by Nourwali I, complications were observed.

## Statistical analysis:

The recorded data was compiled and entered in a spreadsheet computer program (Microsoft Excel 2019) and then exported to data editor page of SPSS version 15 (SPSS Inc., Chicago, Illinois, USA). Quantitative variables were described as means and standard deviations or median and interquartile range based on their distribution. Qualitative variables were presented as count and percentages. For all tests, confidence level and level of significance were set at 95% and 5% respectively.

## Results:

Table 1 shows study participants' genders and ages. The average age of Group 1 members was 34.98 ± 8.92 years, while Group 2 individuals were 38.01 ± 8.01 years. Group 3 had an average age of 37.03 ±9.98 years. (p>0.05) In the present study there were 41, 42 and 38 males respectively in Group 1,2 and 3 respectively while there were 29, 28 and 32 females respectively in above mention groups. Day 1 wound characteristics and general problems are shown in Table 2. Differences were significant between groups in bleeding (p-value 0.001). Group 3 patients had the highest incidence of bleeding, while group 1 had the lowest. Group 3 experienced the highest levels of pain and swelling, while group 1 reported the lowest levels. Disruption of gut flora was statistically significant (p=0.01). Parameters tested on days 4 and 7, as shown in Table 3 and Table 4, exhibit similar trends to day 1. Notably, there are significant differences observed (p ≤ 0.05). Table 5 presents the cost-effectiveness of the therapies that were tested. There were significant cost differences found between Groups 1, 2 and 3 (p ≤ 0.01), according to ANOVA with Tukey HSD Post-hoc Test. Research indicates that synbiotics may be a more effective option than antibiotics when comparing various groups.

## Discussion:

Tooth extraction is a routine procedure that often takes place during oral surgery. However, the use of antibiotics in this context has not been thoroughly investigated. Our study sought to investigate the potential benefits of using synbiotics as a preventive measure to minimise complications following extraction procedures. This approach could potentially lead to a decrease in antibiotic usage when combined with appropriate aseptic techniques. Probiotics have been found to create oral biofilms that can provide protection against dental caries and periodontitis, two common diseases. According to our findings, synbiotics have been shown to offer anti-inflammatory and antimicrobial benefits that are comparable to antibiotics. Additional research is necessary to validate these findings by utilising larger sample sizes and exploring a range of formulations for clinical applications [17, 18-19]. Our study sought to assess the efficacy of synbiotics in preventing local complications following tooth extraction. This approach has the potential to reduce the reliance on antibiotics, provided that proper aseptic techniques are followed during the procedure. Extensive research has shown that probiotics offer a wide range of benefits for overall health and oral well-being. Oral biofilms have the ability to develop in the mouth, acting as a shield for oral tissues against a range of diseases including dental caries, periodontal diseases, halitosis, and candidiasis. This biofilm possesses an extraordinary capability to hinder the colonisation of oral tissues by bacterial pathogens. It also engages in intense competition with cariogenic and periodontal pathogens for resources, significantly limiting their ability to thrive [20, 21-22]. Our research indicates that this synbiotics preparation shows promising potential for improving oral health. It seems to possess properties that could potentially contribute to its therapeutic potential, including anti-inflammatory and antimicrobial effects. Numerous studies have delved into the effects of probiotics and prebiotics on oral health. Research has indicated that probiotics may help reduce symptoms of gingivitis.

Maekawa *et al.* (2014) [23] found evidence supporting this claim, while DN Della Riccia *et al.* (2007) [23] highlighted the anti-inflammatory properties of probiotics and their potential in treating mouth ulcers and oral wounds. The response of the synbiotics group was comparable to that of the antibiotics group within the three groups. This study uses a novel way to compare post-extraction wound healing and indicates synbiotics' benefits. This research should be replicated with more samples and varied formulations for clinical application [24, 25-26]. Since this was a novel approach in comparing post extraction wound healing and as the results showed a definite positive effect by synbiotics. The limitation of the present study is that larger sample size is necessary to increase the strength of this inference. Since the study is a single institution-based descriptive cross-sectional study, the results might not be completely generalizable in other settings. So, a study design with a higher level of evidence is recommended for future studies.

## Source of Funding:

There was no financial support concerning this work.

## Conclusion:

Synbiotics are equally effective as antibiotics in reducing local complications. The effect of synbiotics in promoting oral health, including post-surgical recovery and overall oral well-being is reported.

## Figures and Tables

**Table 1:** Age and gender distribution of the study participants

**Variable**	**GROUP 1**		**GROUP 2**		**GROUP 3**		**Chi square**	**P-Value**
	**N**	**%**	**N**	**%**	**N**	**%**		
**Gender**								
Male	41	58.57	42	59.15	38	54.28	2.47	0.53
Female	29	41.45	28	40	32	45.71		
	**Mean**	**SD**	**Mean**	**SD**	**Mean**	**SD**	**Anova test**	**P value**
Age	34.98	8.92	38.01	8.01	37.03	9.98	2.06	0.31

**Table 2:** Comparison of wound metrics and overall problems between research groups at day 1

**Variable**	**GROUP 1**		**GROUP 2**		**GROUP 3**		**Chi square**	**P value**
	**Present**	**Absent**	**Present**	**Absent**	**Present**	**Absent**		
Bleeding	20	50	28	42	40	30	15	0.001*
Pain	22	48	30	40	35	35	6.5	0.05
Swelling	10	60	18	52	32	38	21.5	0.010*
Gut flora	2	68	8	62	12	58	1.2	0.010*
*Statistically significant

**Table 3:** Comparison of wound metrics and overall problems between research groups at day 4

**Variable**	**GROUP 1**		**GROUP 2**		**GROUP 3**		**Chi square**	**P value**
	**Present**	**Absent**	**Present**	**Absent**	**Present**	**Absent**		
Bleeding	2	68	5	65	8	62	1.5	0.47
Pain	3	67	4	66	12	58	2.1	0.35
Swelling	1	69	3	67	7	63	0.85	0.65
Gut flora	1	69	10	60	14	56	5.5	0.05*

**Table 4:** Comparison of wound metrics and overall problems between research groups at day 7

**Variable**	**GROUP 1**		**GROUP 2**		**GROUP 3**		**Chi square**	**P value**
	**Present**	**Absent**	**Present**	**Absent**	**Present**	**Absent**		
Bleeding	0	70	0	70	0	70	0	1
Pain	0	70	0	70	2	68	0.09	0.89
Swelling	0	70	0	70	0	70	0.19	0.8
Gut flora	0	70	5	65	1	69	0.32	0.72

**Table 5:** Cost analysis during the treatment among the study subjects

**Groups**	**Mean (in Rs)**	**SD**
Group 1	90.27	3.11
Group 2	256.45	1.99
Group 3	29.36	2.46
Anova test	95.63	
P value	<0.01*	
* Statistically significant
ANOVA with Tukey HSD Post-hoc Test
